# Skeletal Muscle-specific PGC-1α Overexpression Suppresses Atherosclerosis in Apolipoprotein E-Knockout Mice

**DOI:** 10.1038/s41598-019-40643-1

**Published:** 2019-03-11

**Authors:** Yuki Shimba, Hanako Togawa, Nanami Senoo, Masahiko Ikeda, Noriyuki Miyoshi, Akihito Morita, Shinji Miura

**Affiliations:** 1 Laboratory of Nutritional Biochemistry, Graduate School of Nutritional and Environmental Sciences, University of Shizuoka, 52-1 Yada, Suruga-ku, Shizuoka 422-8526 Japan; 2 Research and Development Department, Tokai Hit Co., Ltd., 306-1 Gendoji-cho, Fujinomiya-shi, Shizuoka 418-0074 Japan; 3 Faculty of Social and Environmental Studies, Fuji Tokoha University, 325 Ohbuchi, Fuji-shi, Shizuoka 417-0801 Japan; 4 Research Fellow of Japan Society for the Promotion of Science, 5-3-1 Kojimachi, Chiyoda-ku, Tokyo 102-0083 Japan; 5 Graduate School of Environment and Disaster Research, Tokoha University, 325 Ohbuchi, Fuji-shi, Shizuoka 417-0801 Japan; 6 Laboratory of Biochemistry, Graduate School of Nutritional and Environmental Sciences, University of Shizuoka, 52-1 Yada, Suruga-ku, Shizuoka 422-8526 Japan

## Abstract

Endurance exercise training prevents atherosclerosis. Peroxisome proliferator-activated receptor γ coactivator-1α (PGC-1α) increases myokine secretion from the skeletal muscle, and these myokines have been shown to affect the function of multiple organs. Since endurance exercise training increases PGC-1α expression in skeletal muscles, we investigated whether skeletal muscle-specific PGC-1α overexpression suppresses atherosclerosis. Apolipoprotein E-knockout (ApoE-KO)/PGC-1α mice, which overexpress PGC-1α in the skeletal muscle of ApoE-KO mice, were sacrificed, and the atherosclerotic plaque area, spontaneous activity, plasma lipid profile, and aortic gene expression were measured. Immunohistochemical analyses were also performed. The atherosclerotic lesions in ApoE-KO/PGC-1α mice were 40% smaller than those in ApoE-KO mice, concomitant with the reduction in vascular cell adhesion molecule-1 (VCAM-1) and monocyte chemoattractant protein-1 (MCP-1) mRNA and protein levels in the aorta. Spontaneous activity and plasma lipid profiles were not changed by the overexpression of PGC-1α in the skeletal muscle. In human umbilical vein endothelial cells, Irisin and β-aminoisobutyric acid (BAIBA), PGC-1α-dependent myokines, inhibited the tumor necrosis factor α-induced VCAM-1 gene and protein expression. BAIBA also inhibited TNFα-induced *MCP-1* gene expression. These results showed that the skeletal muscle-specific overexpression of PGC-1α suppresses atherosclerosis and that PGC-1α-dependent myokines may be involved in the preventive effects observed.

## Introduction

It is well established that atherosclerosis is one of the principal causes of cardiovascular diseases (CVDs) and stroke^1^. Extensive research has confirmed that endurance exercise training reduces the risk of CVDs and stroke. In epidemiologic studies, moderate physical activity reduces the relative risk of cerebral stroke^2^, and the risk of fatal CVDs was reduced in a 12–22 metabolic equivalents (METS)·h/week physical activity population^3^. The preventive effects of exercise have also been demonstrated in atherosclerosis-prone murine models. For example, running activity over a 12-week period suppressed atherosclerosis progression in apolipoprotein E-knockout mice (ApoE-KO mice)^4^ and low density lipoprotein receptor-knockout mice^5^. Several mechanisms of exercise-mediated prevention of atherosclerosis have been demonstrated^6^. For example, shear stress-induced nitric oxide (NO) generation relaxed vascular smooth muscle cells and improved arterial stiffness^7^. Exercise training improved the function of high density lipoprotein (HDL) and HDL improvement lead to activate NO generation in endothelial cells^8^. Exercise-mediated upregulation of antioxidative enzymes, such as superoxide dismutase, in endothelial cells was also involved in the preventive effect^9^.

Endurance exercise training upregulates peroxisome proliferator-activated receptor γ coactivator-1α (PGC-1α) in the skeletal muscle^10,11^. PGC-1α is a nuclear receptor coactivator expressed in the brown adipose tissue, skeletal muscle, heart, kidney, and brain^12^. We generated transgenic mice overexpressing the PGC-1α-b isoform of PGC-1α in the skeletal muscle (PGC-1α mice)^13,14^. Using the PGC-1α mouse, we revealed that skeletal muscle-specific PGC-1α-b overexpression induced mitochondrial biosynthesis, fiber type switching to oxidative fibers, fatty acid transportation and oxidation, and increased physical endurance^13^. It has been reported that myokines, hormone-like peptides and cytokines which are secreted from skeletal muscle, are involved in the beneficial effects of exercise^15^. For example, interleukin-6 (IL-6) released in response to muscle contraction increases glucose uptake and fatty acid oxidation^16,17^. Brain-derived neurotrophic factor (BDNF), which regulates neuronal survival and growth^18^, is also secreted from the skeletal muscle^19^. Skeletal muscle-specific overexpression of PGC-1α has been shown to stimulate the secretion of myokines, such as Irisin^20^ and β-aminoisobutyric acid (BAIBA)^21^. PGC-1α overexpression in skeletal muscle increased production of FNDC5, a precursor form of Irisin, and Irisin stimulated transformation of white adipose tissue to brown adipose tissue-like characteristics^20^. BAIBA was also reported to induce browning of white adipocyte and stimulate hepatic β oxidation^21^. In an epidemiologic study, it was reported that there is a positive correlation between serum Irisin levels and flow-mediated dilatation (FMD), an independent predictor of atherosclerosis^22^. Furthermore, Lee and colleagues reported that serum Irisin was a significant independent predictor for carotid atherosclerosis in peritoneal dialysis patients^23^.

We hypothesized that overexpression of PGC-1α in skeletal muscles may suppress atherosclerosis via increased secretion of myokines into the blood stream. In this study, we investigated whether skeletal muscle-specific PGC-1α overexpression suppresses atherosclerosis using a murine ApoE-KO model.

## Results

### Decrease in atherosclerosis lesions by overexpression of PGC-1α in skeletal muscle

**Figure 1:**
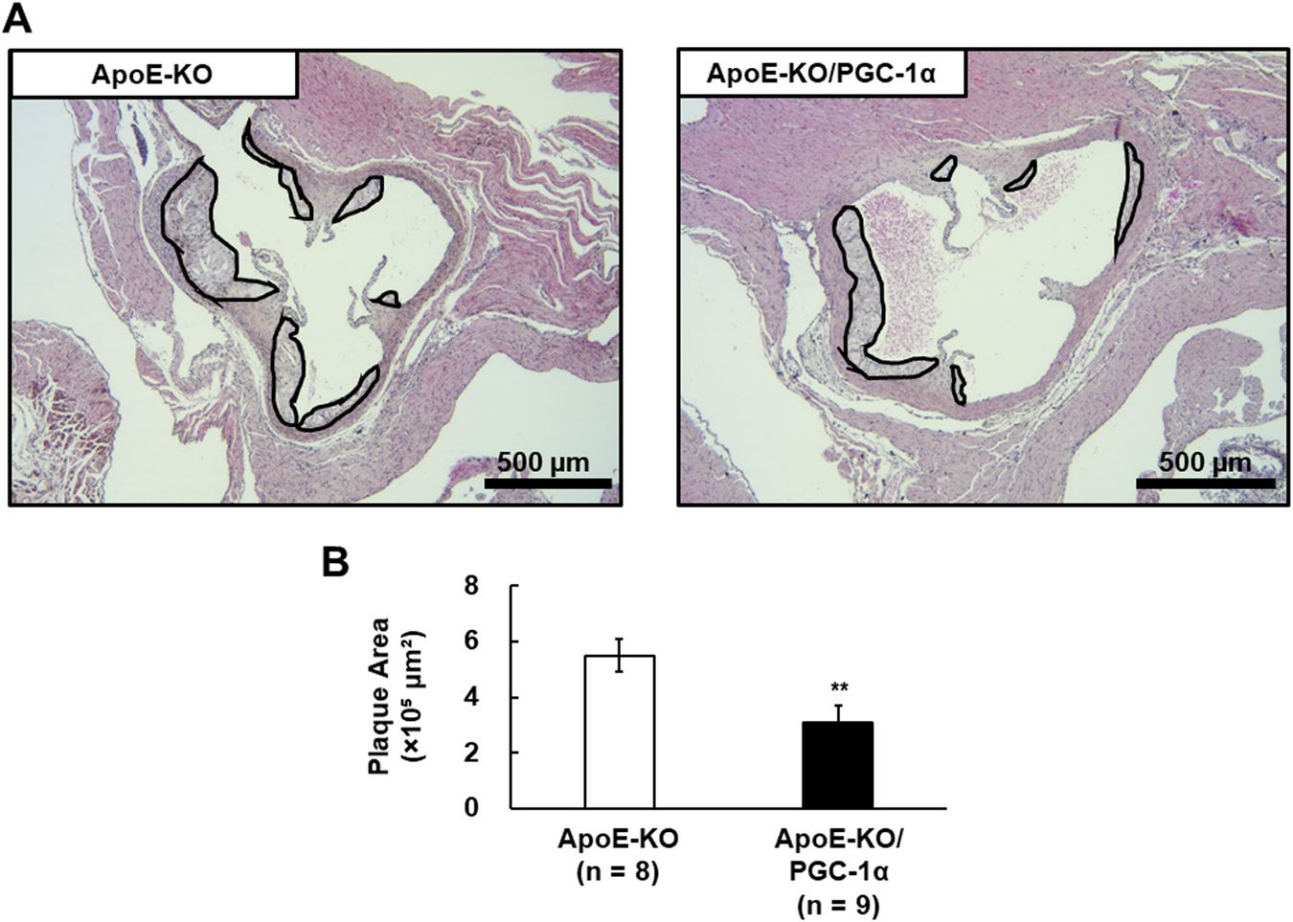
Influence of skeletal muscle-specific PGC-1α overexpression on atherosclerotic plaque formation in ApoE-KO mice. (**A**) Representative images of H&E staining of the aortic valve from ApoE-KO and ApoE-KO/PGC-1α mice. The area surrounded by a black line shows atherosclerotic plaques. (**B**) Quantitation of plaque area. The data are expressed as the mean ± SEM. **p < 0.01 vs. ApoE-KO mice.

**Figure 2:**
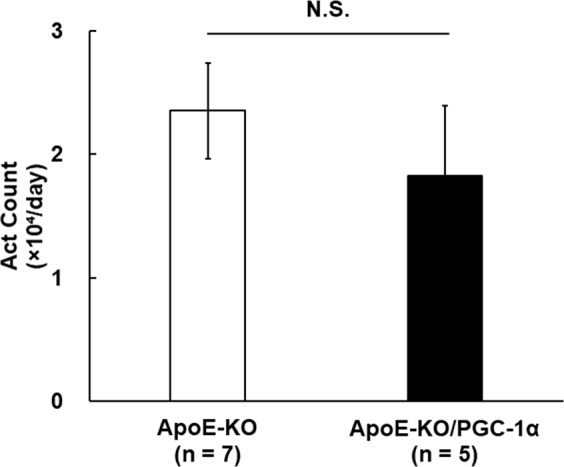
Spontaneous activity in ApoE-KO mice and ApoE-KO/PGC-1α mice. Spontaneous activity in ApoE-KO mice and ApoE-KO/PGC-1α mice is shown. There were no significant differences between the two groups of mice. N.S.: no significant. The data are expressed as the mean ± SEM.

We generated ApoE-KO/PGC-1α mice, overexpressing PGC-1α-b in the skeletal muscle of ApoE-KO mice. Since plasma triglyceride (TG) and total cholesterol (TC) levels are increased greatly and atherosclerosis is initiated in ApoE-KO mice fed with normal chow^24^, we fed ApoE-KO mice normal chow. The mean body weight of the ApoE-KO mice was 31.1 ± 0.7 g and that of ApoE-KO/PGC-1α mice was 27.9 ± 1.8 g, indicating that PGC-1α overexpression in skeletal muscle did not affect body weight. Representative photomicrographs of hematoxylin and eosin (H&E)-stained aorta sections from ApoE-KO and ApoE-KO/PGC-1α mice are presented in Fig. 1A. Atherosclerotic plaque areas were smaller in the aortic sections of ApoE-KO/PGC-1α mice than in ApoE-KO mice. Quantification of the aortic plaque areas showed that these were significantly lower in ApoE-KO/PGC-1α mice, approximately 40% on average compared with those in ApoE-KO mice (3.1 ± 0.2 × 10^5^ μm^2^ versus 5.5 ± 0.6 × 10^5^ μm^2^, *p* < 0.01; Fig. 1B). Such plaques were not formed in wild-type C57BL6/J (WT) mice (data not shown). Spontaneous activity of ApoE-KO mice was not significantly changed by the overexpression of PGC-1α (Fig. 2). Thus, skeletal muscle-specific overexpression of PGC-1α suppressed atherosclerosis progression in ApoE-KO mice without increasing spontaneous activity. The plaque areas of aged ApoE-KO/PGC-1α mice (37–41 weeks old) were also significantly lower in comparison to those in aged ApoE-KO mice (Supplemental Fig. S1).

### Effects of overexpression of PGC-1α on plasma lipid profiles

In order to reveal how skeletal muscle-specific PGC-1α overexpression suppressed the progression of atherosclerosis, we measured the plasma lipid profiles of mice because dyslipidemia is one of the leading high-risk factors for atherosclerotic disease^25^. Plasma lipid profiles are shown in Table 1. Only plasma chylomicron cholesterol concentrations were higher in ApoE-KO/PGC-1α mice than in ApoE-KO mice. However, there were no differences between the two groups with respect to other lipoproteins. Since it is reported that chylomicron cholesterol does not affect the progress of atherosclerosis^26^, these results showed that overexpression of PGC-1α in the skeletal muscle did not affect atherosclerosis-related plasma lipid concentrations. We measured the levels of plasma thiobarbituric acid reactive substance (TBARS), a biological marker of oxidative stress^27^. The reduction in plasma TBARS levels was not observed by the overexpression of PGC-1α in ApoE-KO mice (Supplemental Fig. S2). Blood glucose levels under fasting, one of the major risk factors for atherosclerosis^28^, were also measured in ApoE-KO/PGC-1α mice. Fasting blood glucose levels were not significantly different between ApoE-KO/PGC-1α mice and ApoE-KO mice (Supplemental Fig. S3). Although previous studies showed that plasma sphingomyelin (SM) correlated with the development of coronary artery diseases^29^, plasma SM concentrations were not lowered by the overexpression of PGC-1α in ApoE-KO mice (Supplemental Fig. S4).

**Table 1:** Plasma lipid levels in ApoE-KO and ApoE-KO/PGC-1α mice.

Parameters (mg/dL)	ApoE-KO (n = 6)	ApoE-KO/PGC-1α (n = 8)	Significance
TC	483 ± 46	572 ± 33	n.s.
CM-C	67 ± 7	91 ± 7	*
VLDL-C	312 ± 31	379 ± 25	n.s.
LDL-C	87 ± 8	84 ± 4	n.s.
HDL-C	17 ± 3	17 ± 1	n.s.
Sd-LDL-C	29 ± 3	28 ± 1	n.s.
Non-HDL-C	466 ± 44	555 ± 33	n.s.
TG	58 ± 7	57 ± 8	n.s.
CM-TG	15 ± 2	16 ± 2	n.s.
VLDL-TG	31 ± 4	30 ± 5	n.s.
LDL-TG	8.9 ± 0.7	8.5 ± 1.3	n.s.
HDL-TG	2.9 ± 0.4	2.7 ± 0.3	n.s.

TC, total cholesterol; CM-C, chylomicron-cholesterol; VLDL-C, very low density lipoprotein-cholesterol; LDL-C, low density lipoprotein-cholesterol; HDL-C, high density lipoprotein-cholesterol; Sd-LDL-C, small dense-LDL-cholesterol; TG, triglyceride; CM-TG, chylomicron-triglyceride; VLDL-TG, very low density lipoprotein-triglyceride; LDL-TG, low density lipoprotein-triglyceride; HDL-TG, high density lipoprotein-triglyceride. *p < 0.05 vs. ApoE-KO mice. The data are expressed as the mean ± SEM.

### Changes in mRNA expression in the aorta by overexpression of PGC-1α in skeletal muscle

**Figure 3:**
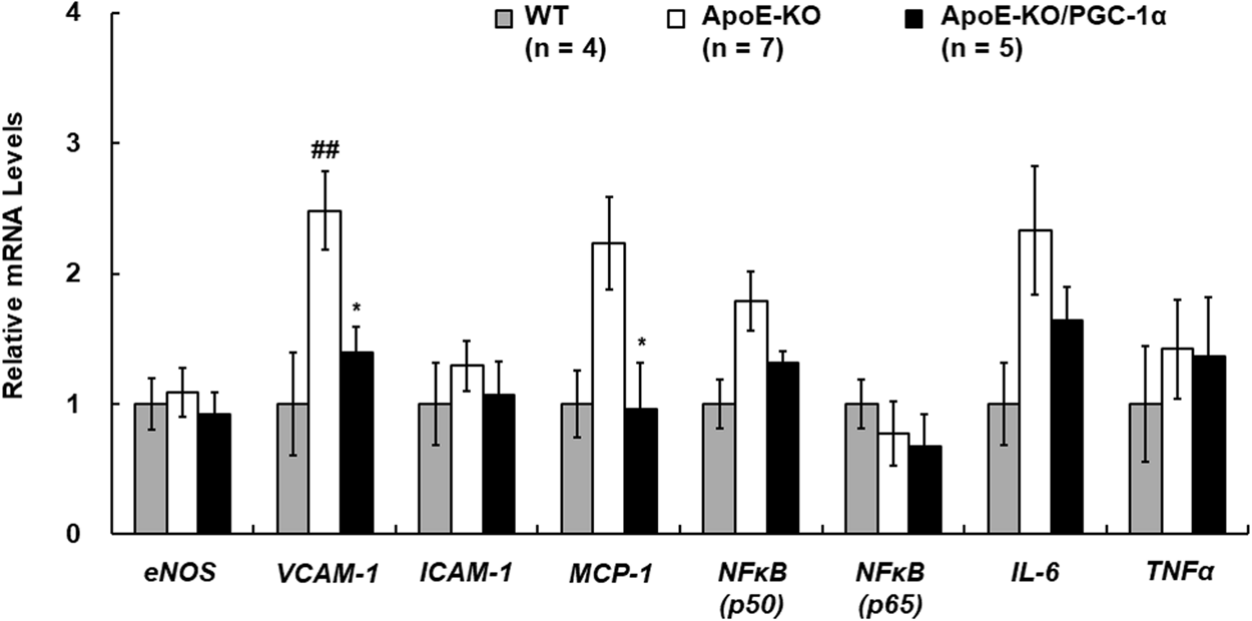
Aortic gene expression in wild-type (WT), ApoE-KO, and ApoE-KO/PGC-1α mice. Relative mRNA expression levels of *eNOS*, *VCAM-1*, *ICAM-1*, *MCP-1*, *NFκB* (*p50*, *p65*), *IL-6*, and *TNFα* in the abdominal aorta were measured by RT-qPCR (4–7 mice in each group). Amplification of *β-actin* mRNA was used to normalize for differences in RNA extraction and amplification. The data are expressed as fold of the mean values in the WT group. Values are presented as the mean ± SEM. ^##^*p* < 0.01 vs. WT mice. *p < 0.05 vs. ApoE-KO mice.

Inflammatory mediators have been shown to be involved in the progression of atherosclerosis^30^. Vascular cell adhesion molecule-1 (VCAM-1) and intercellular adhesion molecule-1 (ICAM-1) mediate monocyte adhesion on endothelial cells^31^. Monocyte chemoattractant protein-1 (MCP-1) mediates monocyte migration underneath endothelial cells^32^. Nuclear factor kappa B (NFκB), which consists of two subunits (p50, p65), regulates the expression of VCAM-1, ICAM-1, and MCP-1^33,34^. Interleukin-6 and tumor necrosis factor α (TNFα), are inflammatory cytokines and are expressed in plaques^35,36^. The changes in mRNA levels of these inflammatory mediators in the abdominal aorta are shown in Fig. 3. In ApoE-KO mice, *VCAM-1* mRNA expression was significantly higher compared to WT mice as reported previously^37^. However, *VCAM-1* and *MCP-1* mRNA expression levels in the aorta from ApoE-KO/PGC-1α mice were significantly lower compared to that in ApoE-KO mice. On the other hand, although mRNA expression of inflammatory mediators in peripheral blood cells was reported as an indicator for inflammatory status and the disease state^38^, no significant differences were observed in the mRNA expression of inflammatory mediators in blood cells among three groups of mice (Supplemental Fig. S5). These data suggested that PGC-1α overexpression in skeletal muscle suppressed *VCAM-1* and *MCP-1* mRNA expression in the aorta, and that *VCAM-1* and *MCP-1* suppression might be involved in prevention of plaque formation, particularly because inflammatory processes play key roles in the progression of atherosclerosis^39^.

### Immunohistochemical analyses of aorta cryosections from ApoE-KO/PGC-1α mice

**Figure 4:**
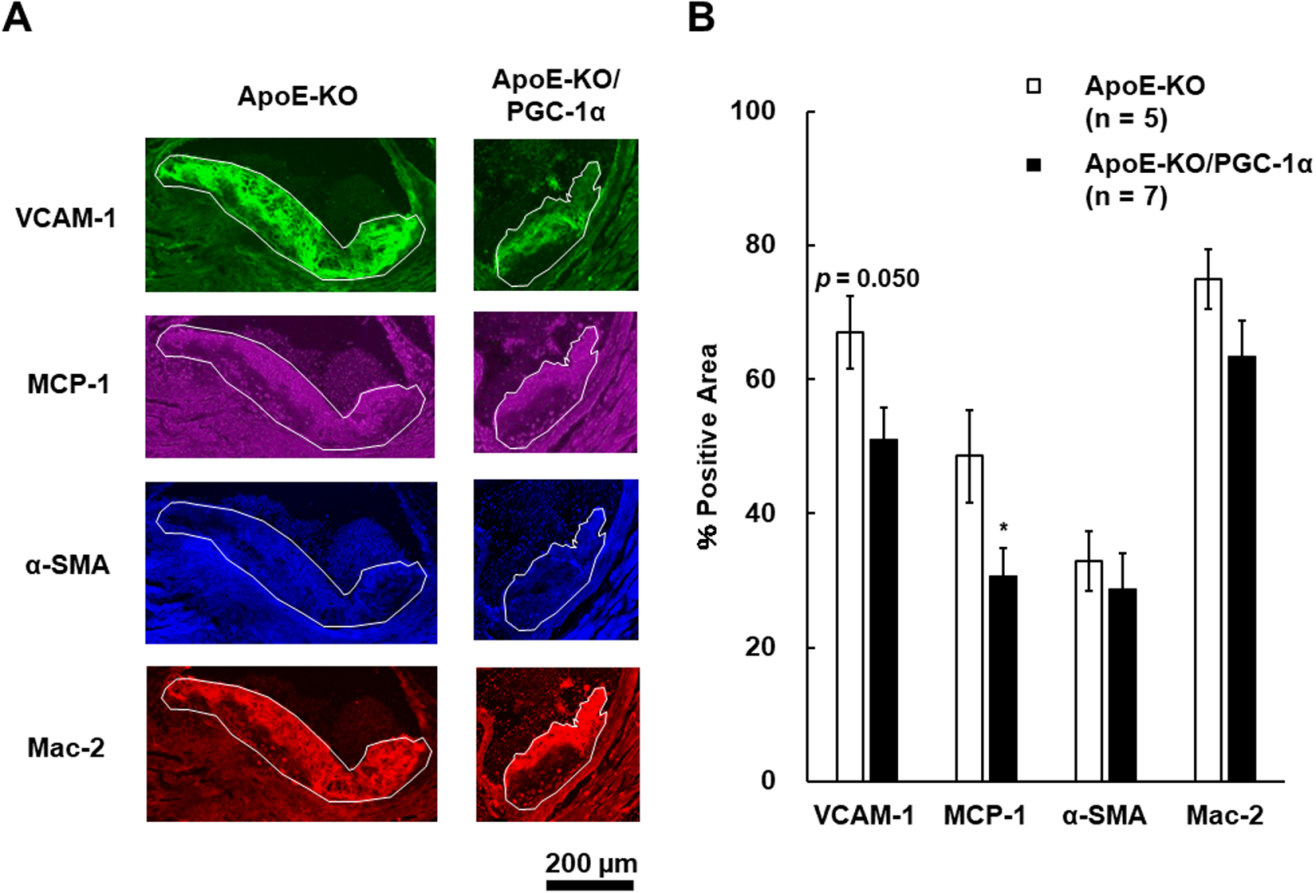
Immunohistochemical analyses of atherosclerotic plaques in aortic valve. (**A**) Representative images of aortic valve sections stained with anti-VCAM-1, anti-MCP-1, anti-α-SMA, and anti-Mac-2 antibodies. The white line shows plaque area. (**B**) Quantitative analysis of stained areas. Positive-staining areas were divided by total plaque area and results are presented as % positive area of the total. The data are expressed as the mean ± SEM. *p < 0.05 vs. ApoE-KO mice.

Since the suppression of *VCAM-1* and *MCP-1* mRNA expression was identified in the abdominal aorta from ApoE-KO/PGC-1α mice, immunohistochemical analyses were performed to determine whether VCAM-1 and MCP-1 protein abundance were also suppressed in atherosclerotic plaque areas around aortic valves. Multiple photomicrographs obtained after immunofluorescent staining are shown in Fig. 4. The positive staining areas for VCAM-1 and MCP-1 in ApoE-KO/PGC-1α mice were lower compared with those in ApoE-KO mice. Thus, skeletal muscle-specific PGC-1α overexpression suppressed VCAM-1 and MCP-1 protein expression in atherosclerotic plaques around aortic valves. Vascular smooth muscle cells are regarded as exerting a plaque-stabilization effect^40^. Macrophages are differentiated from monocytes and involved in atherosclerosis progression^39^. Therefore, we also analyzed vascular smooth muscle proliferation and macrophage localization using immunofluorescent staining with anti-α-smooth muscle actin (α-SMA) and anti-Galectin 3 (Mac-2) antibodies. However, α-SMA positive smooth muscle cells and Mac-2 positive macrophages were not significantly altered by skeletal muscle-specific overexpression of PGC-1α in ApoE-KO mice.

### PGC-1α-mediated changes of gene expression involved in the production of Irisin and BAIBA in the skeletal muscle of ApoE-KO mice

**Figure 5:**
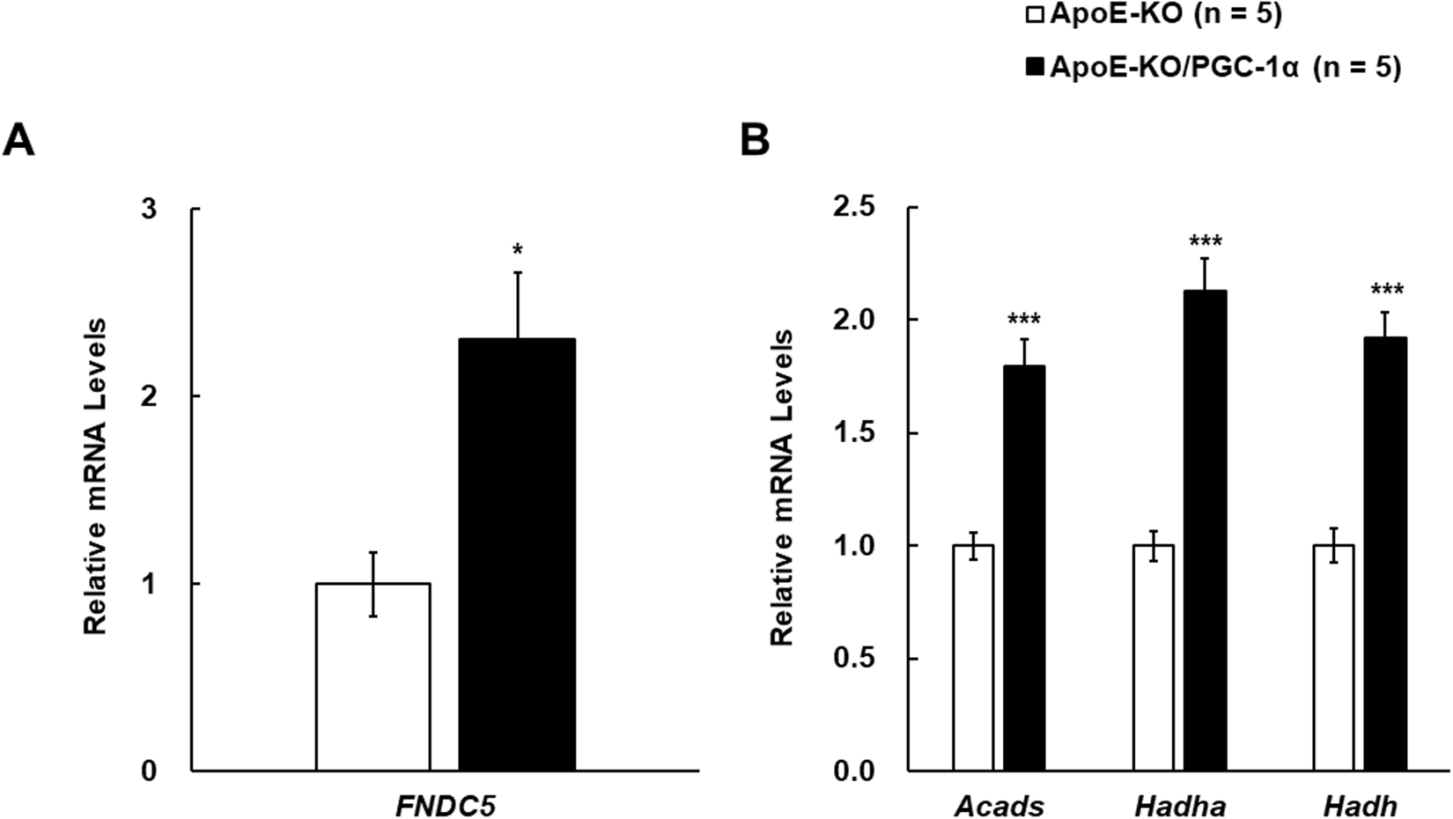
Gene expression level of *FNDC5* and enzymes required for BAIBA biosynthesis in the muscle of ApoE-KO and ApoE-KO/PGC-1α mice. (**A**) Relative mRNA expression levels of *FNDC5* in the muscle of the two groups were measured by RT-qPCR (4–7 mice in each group). (**B**) Relative mRNA expression levels of *Acads*, *Hadha*, and *Hadh* in the muscle of ApoE-KO and ApoE-KO/PGC-1α mice. Amplification of *β-actin* mRNA was used to normalize for differences in RNA extraction and amplification. The data are expressed as fold of the mean values in the ApoE-KO group. Values are presented as the mean ± SEM. *p < 0.05, ***p < 0.001 vs. ApoE-KO mice.

Irisin and BAIBA are known as PGC-1α-dependent myokines^20,21^. To determine whether PGC-1α-mediated production of these myokines was observed in ApoE-KO mice, we measured the expression levels of genes involved in biosynthesis of Irisin and BAIBA. As shown in Fig. 5A, *FNDC5*, which is the gene symbol of Irisin, was increased 2.3-fold in the muscle of ApoE-KO/PGC-1α mice. mRNA required for BAIBA biosynthesis, such as *Acyl-CoA dehydrogenase short chain* (*Acads*), *hydroxyacyl-CoA dehydrogenase trifunctional multienzyme complex subunit alpha* (*Hadha*), and *hydroxyacyl-Coenzyme A dehydrogenase* (*Hadh*) were increased in ApoE-KO/PGC-1α mice compared to ApoE-KO mice (Fig. 5B). Therefore, skeletal muscle-specific PGC-1α overexpression increased the expression of genes involved in production of Irisin and BAIBA in the muscles of ApoE-KO mice.

### Effect of Irisin and BAIBA, PGC-1α-dependent myokines, on VCAM-1 and MCP-1 expression in human umbilical vein endothelial cells (HUVECs)

**Figure 6:**
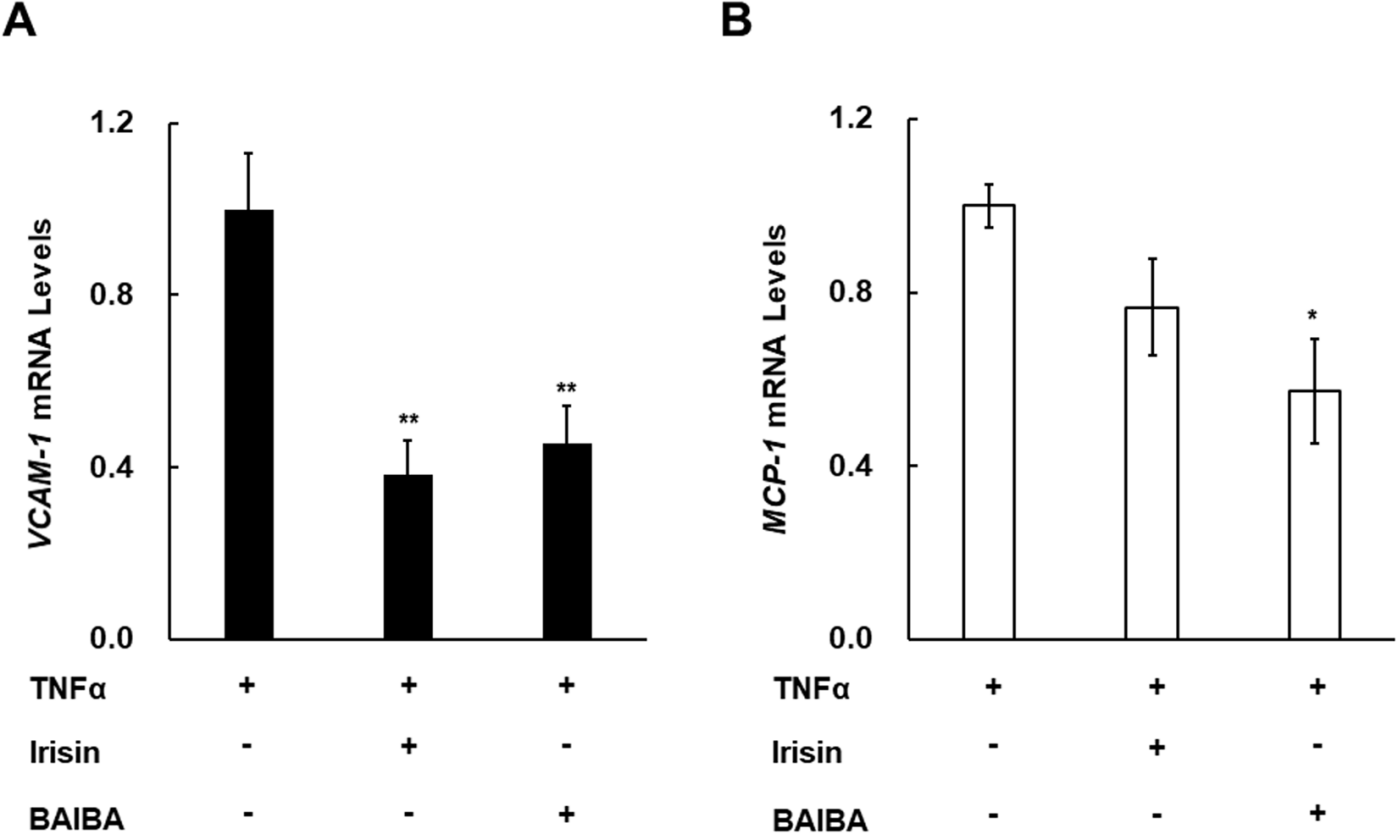
Effects of Irisin and BAIBA on TNFα-induced *VCAM-1* and *MCP-1* gene expression in HUVECs. (**A**) *VCAM-1* gene expression in HUVECs was measured after treatment with Irisin or BAIBA in the presence of TNFα. (**B**) *MCP-1* gene expression in HUVECs was measured after treatment with Irisin or BAIBA in the presence of TNFα. Expression of these mRNAs was measured by RT-qPCR (n = 6). Amplification of *β-actin* mRNA was used to normalize for differences in RNA extraction and amplification. The data are expressed relative to the mean value in the TNFα treated HUVECs. Values are the relative mean ± SEM. *p < 0.05, **p < 0.01 vs. TNFα-treated cells.

PGC-1α overexpression in the skeletal muscle suppressed VCAM-1 and MCP-1 expression in the aorta; however, it is unclear how VCAM-1 and MCP-1 were suppressed. We hypothesized that PGC-1α-dependent production and secretion of myokines might suppress VCAM-1 and MCP-1 expression in endothelial cells. Therefore, we exposed TNFα-treated HUVECs to Irisin and BAIBA because TNFα is involved in atherosclerosis progression^41,42^. TNFα, for instance, is present in atherosclerotic arterial walls in humans^36^ and administration of TNFα to ApoE-KO mice promotes the progression of atherosclerosis^42^. Irisin and BAIBA reduced TNFα-induced mRNA expression of *VCAM-1* in HUVECs (Fig. 6A). *MCP-1* mRNA expression was also decreased by addition of BAIBA (Fig. 6B). The effect of the myokines on the expression of other genes related to atherogenesis is shown in Supplemental Fig. S6.

**Figure 7:**
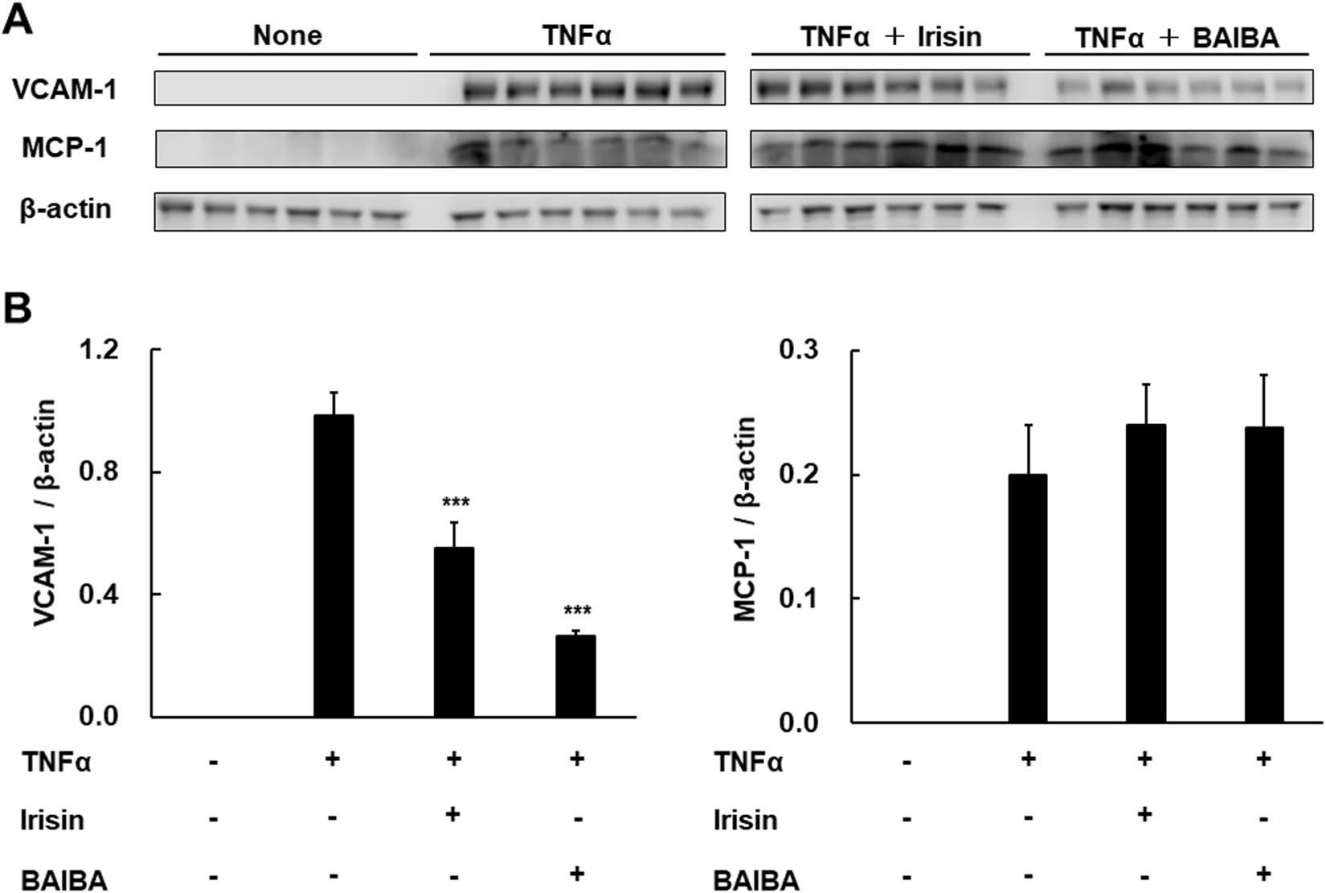
Effects of Irisin and BAIBA on TNFα-induced VCAM-1 and MCP-1 protein expression in HUVECs. (**A**) HUVECs were treated with Irisin or BAIBA in the presence or absence of TNFα. Protein abundance of VCAM-1 and MCP-1 was assessed by western blotting. (**B**) Quantitation of VCAM-1 and MCP-1 from (**A**), normalized to β-actin. The samples derive from the same experiment and blots were processed in parallel. Full-length blots are presented in Supplementary Fig. S7. The data are expressed relative to the mean ratio to β-actin protein expression. Values are relative mean ± SEM. ***p < 0.001 vs. TNFα treated cells.

In order to confirm whether Irisin and BAIBA suppress VCAM-1 and MCP-1 protein expression as well as gene expression, we also measured protein expression levels directly. Incubation of the cells with TNFα increased VCAM-1 and MCP-1 protein expression significantly. Irisin and BAIBA suppressed TNFα-induced VCAM-1 protein expression in HUVECs (Fig. 7). Therefore, Irisin and BAIBA decreased the expression of VCAM-1 at the level of both mRNA and protein production.

## Discussion

The present study shows that skeletal muscle-specific PGC-1α overexpression suppressed the progression of atherosclerosis in ApoE-KO mice without changing spontaneous activity and plasma lipid profiles. Overexpression of PGC-1α in skeletal muscle suppressed VCAM-1 and MCP-1 expression in the aorta. Furthermore, Irisin and BAIBA suppressed TNFα-induced VCAM-1 mRNA and protein expression in HUVECs.

Plasma lipid profiles were not changed by muscular overexpression of PGC-1α in ApoE-KO mice. Previous studies showed that improvement of plasma lipid profiles was not necessary for the suppression of atherosclerosis by endurance exercise training in mouse models. In the report by Pellegrin, 24 weeks of swimming exercise decreased the progression of atherosclerotic lesions by 30% in ApoE-KO mice without changes to plasma TG, TC, HDL-cholesterol, non-HDL-cholesterol, and phospholipid levels^43^. Eight weeks of swimming also suppressed fatty streak plaque lesions developed in ApoE-KO mice fed a high-fat diet without changing plasma TG and TC levels^44,45^. These previous studies suggested that exercise-induced antioxidant effects via the vascular NO system suppressed atherosclerosis. However, plasma TBARS levels and mRNA expression of *eNOS* in the aorta were not improved in 16 to 20-week-old ApoE-KO mice by overexpression of PGC-1α in skeletal muscles, suggesting that plasma lipid profiles and antioxidant systems were not involved in the suppression of atherosclerosis observed in ApoE-KO/PGC-1α mice. Exercise is recommended to lower LDL-cholesterol and non-HDL-cholesterol^46^ for the prevention of atherosclerotic disease. However, exercise therapy appears to suppress atherosclerosis even when there is no change in plasma lipid profiles.

In addition to lipid profiles and antioxidant systems, chronic arterial wall inflammation is a key component of atherosclerosis^47^. VCAM-1, ICAM-1, MCP-1, NFκB, IL-6 and TNFα are involved in atherogenesis^30^. Among these factors, VCAM-1 and MCP-1 have been reported as factors for the initiation of atherosclerosis^31,32^. VCAM-1 induces monocyte adhesion and accumulation on the vessel wall, and MCP-1 induces monocyte migration and infiltration under the vessel wall, respectively^31,32^. Requirement of VCAM-1 for the initiation of atherosclerosis was shown using a *Vcam1*^D4D^/LDL receptor-knockout mouse model^48^. In addition, MCP-1 deficiency was reported to suppress the progression of atherosclerosis in ApoE-KO mice^49^. Therefore, decreased expression of VCAM-1 and MCP-1 suppresses the development of atherosclerosis. Furthermore, the expression of VCAM-1 and MCP-1 in the aorta was reduced by exercise training^50^^,^^51^, suggesting that endurance exercise training might decrease aortic VCAM-1 and MCP-1 expression in preventing atherosclerosis. In the present study, we found that VCAM-1 and MCP-1 expression was lower in the aorta from ApoE-KO/PGC-1α mice. It seems likely that decreases in the expression of these factors are implicated in the suppression of atherosclerosis by the overexpression of PGC-1α in the skeletal muscle.

In this study, Irisin and BAIBA reduced VCAM-1 protein expression in HUVECs, as observed in the aorta of ApoE-KO/PGC-1α mice. However, although MCP-1 protein expression was suppressed in the aorta by skeletal muscle PGC-1α overexpression, TNFα induced expression was not reduced in HUVECs treated with Irisin and BAIBA. In order to rationalize this difference, we considered that the suppression of MCP-1 expression may be occurring in macrophages. VCAM-1 is expressed by endothelium and mediates monocyte adhesion^48^. MCP-1, however, is expressed, not only in endothelial cells, but also in monocytes and macrophages and acts as a potent monocyte chemotactic factor^32^. Therefore, myokines may act on macrophages to suppress the expression of MCP-1. Furthermore, unidentified factor(s) other than Irisin and BAIBA might also involve in the suppression of MCP-1 expression in aorta, *in vivo*.

Recently, it was reported that myokines are secreted from skeletal muscle and affect not only the skeletal muscle itself but also other organs such as the liver and adipose tissue^15^. Epidemiological studies revealed a correlation between Irisin, and CVDs risk^22,23^. Protective effects of Irisin on atherosclerosis were also reported in two different ApoE-KO mouse models^37,52^. In these experiments, Irisin treatment significantly decreased atherosclerotic plaque area concomitantly with the reduction of inflammatory cytokines expression in aorta. Since overexpression of PGC-1α in skeletal muscle increased the production and secretion of Irisin from skeletal muscle^20^, Irisin secretion might represent one of the mechanisms for suppression of atherosclerosis. In the present study, VCAM-1 and MCP-1 expression were lower in the aorta as a result of PGC-1α overexpression in skeletal muscle. Moreover, we found that Irisin treatment of HUVECs suppressed TNFα-induced expression of VCAM-1 mRNA and protein. In addition to Irisin, BAIBA was reported as a myokine and secreted from cells with forced expression of PGC-1α, which results in the browning of white adipose tissue and increases fat oxidation in the liver^21^. Their study also revealed that, in humans, plasma BAIBA levels were increased with exercise and inversely associated with metabolic risk factors, such as fasting glucose, insulin, homeostasis model assessment of insulin resistance (HOMA-IR), TG, and TC levels^21^. We have previously reported that BAIBA was detected only in mice that overexpressed PGC-1α in the skeletal muscle, but not in WT mice^53^. However, the protective effects of BAIBA on the progression of atherosclerosis and inflammatory reactions on the vessel walls have not been uncovered. In the present study, we observed that BAIBA treatment of HUVECs suppressed the TNFα-induced expression of VCAM-1, as also observed for Irisin. The plasma BAIBA concentration was 6.5 ± 2.5 µM in mice overexpressing PGC-1α in skeletal muscle^21^ and we also observed decreased mRNA and protein expression of VCAM-1 at 10 and 40 µM of BAIBA treatment, respectively, in *in vitro* experiments, suggesting that increased plasma BAIBA levels may play an important role in the anti-atherogenic effects of PGC-1α overexpression in skeletal muscle. On the other hand, adiponectin has beneficial effects in terms of atherosclerotic progression^54^. Okamoto *et al*. reported that adenovirus-mediated elevation of plasma adiponectin suppressed atherosclerosis progression in ApoE-KO mice concomitantly with suppression of aortic *VCAM-1* mRNA expression^55^. Irisin enhanced adiponectin expression in lipopolysaccharide-treated adipocytes^56^. Therefore, adiponectin-mediated mechanisms might also be involved in the prevention of atherosclerosis by PGC-1α overexpression in the skeletal muscle.

In this study, we show that atherosclerosis was suppressed in the ApoE-KO/PGC-1α mice, and the mRNA expression levels of *FNDC5* and the genes involved in BAIBA biosynthesis in skeletal muscle were increased about 2-fold in the ApoE-KO/PGC-1α mice. It has been reported that 3 weeks of exercise training could increase plasma myokines concentrations (2.5-fold for Irisin^20^ and 1.2-fold for BAIBA^21^), and exercise training for 20 weeks suppressed atherosclerosis in ApoE-KO mice^4^. Prolonged secretion of these myokines for 20 weeks may be involved in suppression of atherosclerosis in exercise-trained ApoE-KO mice. In humans, endurance training causes a 2-fold increase in the protein expression of PGC-1α in skeletal muscle^57^. Endurance training increases serum Irisin levels 1.2-fold^58^ and plasma BAIBA concentrations by 17%^21^. Continuous training is expected to increase the expression of PGC-1α and the increased levels of these myokines in plasma. Therefore, Irisin and BAIBA might be involved in exercise-induced atherosclerosis suppression in humans in the same manner as in ApoE-KO/PGC-1α mice.

In conclusion, we have shown that PGC-1α overexpression in skeletal muscle suppressed VCAM-1 and MCP-1 expression in the arterial wall and inhibited the progression of atherosclerosis. Furthermore, we demonstrated that PGC-1α-dependent myokines, namely Irisin and BAIBA, reduced TNFα-induced VCAM-1 expression. These findings indicate that adaptive effects of endurance training on the skeletal muscle might be one of the reasons for exercise training-mediated anti-atherogenic effects. Irisin and BAIBA may be useful biomarkers to identify whether endurance exercise training is sufficient to prevent atherosclerotic diseases.

## Materials and Methods

### Animals

Homozygous ApoE-KO mice and heterozygotes PGC-1α mice were crossbred and backcrossed into a murine ApoE-KO background. ApoE-KO mice and homozygous ApoE-KO/heterozygotes PGC-1α-b mice (ApoE-KO/PGC-1α mice) were obtained, and 16–20-week-old male offspring were used in experiments. All mice were maintained on a C57BL6/J background. ApoE-KO mice were obtained from The Jackson Laboratory (Bar Harbor, ME, USA)^59^. The methods for generation of PGC-1α mice were described previously^14^. The promoter for human α-skeletal actin, provided by Drs E. C. Hardeman and K. L. Guven (Children’s Medical Research Institute, Australia) was used to express PGC-1α-b in skeletal muscle. Animals were housed in groups of 5 mice per cage in a room with a 12-hour light/dark cycle at 22 °C and provided with standard mouse chow (CE-2, CREA Japan Inc., Tokyo, Japan) and drinking water *ad libitum*. Mice were cared for according to the National Institutes of Health Guide for the Care and Use of Laboratory Animals (https://www/ncbi.nlm.nih.gov/books/NBK54050/) and our institutional guidelines. All animal experiments were approved by the Institutional Animal Care and Use Committee of the University of Shizuoka (number 165123).

### Spontaneous activity

Nineteen-week-old mice were housed in a single cage equipped with infrared ray sensor (NS-AS01, NeuroScience, Tokyo, Japan) for 24 hours. The data were analyzed using ARCO-2000 (ARCO SYSTEM Inc., Chiba, Japan).

### Fasting blood glucose levels

Mice (16–17 weeks old) were starved for 12 hours before blood sampling. The blood samples were collected from the tail vain. Blood glucose levels were measured using Breeze 2 (Bayer, Leverkusen, Germany).

### Plasma lipid analysis

Mice were starved for 12 hours before blood sampling. The blood samples were collected from the orbital sinus under isoflurane anesthesia. EDTA was used as an anti-coagulant. The plasma was separated and stored at −80 °C until analysis. Plasma lipid profiles were analyzed by LipoSEARCH service (Skylight Biotech, Inc., Akita, Japan)^60^. Thiobarbituric acid reactive substance concentrations in pooled plasma were measured using Calorimetric TBARS Microplate Assay Kit (FR40, Rochester Hills, Oxford Biochemical Research, MI, USA) according to the manufacturer’s instructions. Plasma SM was measured as described previously^61^. In brief, SMs were analyzed by LCMS-8040 (Shimadzu Corp., Kyoto, Japan) under the positive-ion mode using precursor-ion mode scanning at m/z 184 to specifically detect substances containing choline. Obtained MS data were searched with a database of sphingolipids (http://www.lipidmaps.org/tools/ms/sphingolipids_batch.html). The relative peak area for each species was normalized by the peak area of internal standard (1,2-diheptadecanoyl-sn-glycero-3-phosphocholine, 850360 P, Avanti Polar Lipids, Alabaster, AL, USA).

### Determination of aortic lesion area

Hearts were fixed with 10% formalin neutral buffer solution for 72 hours, followed by replacement with phosphate buffered saline (PBS). Cryosections (5-μm-thick) were taken at the four levels of the aortic valves and stained with H&E. The area of the atherosclerotic lesions from 4 cross-sections of each mouse heart was measured using Image J software (http://imagej.nih.gov/ij/). Atherosclerotic lesions were calculated as the sum of lesion area across 4 cross-sections^62^.

### Immunohistochemistry

Cryosections of the aortic valve were fixed on ice (4 °C) with acetone for 5 minutes and washed with 1% Tween in PBS (PBS-T) for 5 minutes 3 times. Cryosections were incubated with Blocking Mouse IgG (MKB-2213, Vector Laboratories, CA, USA) in 1 mL PBS. After washing with PBS-T for 5 minutes 3 times, cryosections were incubated with 5% Normal Goat Serum (50062Z, Thermo Fisher Scientific, Kanagawa, Japan), followed by overnight-incubation with primary antibodies in PBS-T at 4 °C. After incubation, the cryosections were washed with PBS-T for 5 minutes 3 times and incubated with secondary antibodies in PBS-T for 1 hour at room temperature (15–25 °C). After washing with PBS-T for 5 minutes 3 times, sections were mounted with Prolonged Gold (P10144, Invitrogen, Carlsbad, CA, USA). Image acquisitions were performed with a DMi8 inverted microscope (Leica microsystems, Tokyo, Japan) and image analysis was performed using Leica Application Suite X software (Version 3.04.16529, Leica microsystems). Stained areas were divided by total plaque area and results indicated as the percent of positive areas in plaque areas. Staining was performed using mouse anti-VCAM-1 (1:200, #550547, BD bioscience, San Jose, NJ, USA), anti-MCP-1 (1:50, #ab7202, abcam, Cambridge, UK), anti-α-SMA (1:200, #ab7817, abcam), anti-Mac-2 (1:16, #ab2785, abcam) antibodies as primary antibodies for multiple staining. Secondary antibodies included goat anti-mouse IgM Alexa 488 (1:500, A21042, Invitrogen), goat anti-rabbit IgG Alexa 647 (1:200, A21244, Invitrogen), goat anti-mouse IgG2b Alexa 350 (1:200, A21140, Invitrogen), and goat anti-mouse IgG1 Alexa 555 (1:500, A21127, Invitrogen).

### Quantitative reverse transcription-polymerase chain reaction (RT-qPCR)

The aortas were dissected and kept in liquid nitrogen. Total RNA was extracted from aortas using 1 mL of RNA iso plus (9108, Takara Bio Inc., Shiga, Japan). Whole blood was collected and total RNA extracted using RNA iso blood (9112, Takara Bio Inc.). Reverse transcription (RT) was performed with PrimeScript RT reagent Kit with gDNA Eraser (RR047A, Takara Bio Inc.) carried out using 1 µg of RNA. Real-time quantitative PCR (qPCR) was conducted with SYBR Premix Taq II (RR820S, Takara Bio Inc.). RNA extraction, RT, and qPCR were performed according to the manufacturers’ protocols. Primer sequences are listed in Supplemental Table S1. Expression of target genes was normalized to that of the housekeeping *β-actin* gene using the standard curve method. All data are presented as fold-change over that of WT (C57BL6/J) or ApoE-KO mice.

### Gene expression assay in HUVECs

HUVECs (C2519A) were purchased from Lonza Japan (Tokyo, Japan). Cells were cultured in EGM-2 Endothelial Cell Growth Medium-2 BulletKit (CC-3162, Lonza Japan). The cells were used between passages 4 and 6. All experiments were carried out with the same batch of HUVECs, which were from pooled donors. HUVECs were incubated with TNFα (10 ng/mL) (203–15263, Wako Pure Chemical Industries, Ltd., Osaka, Japan), Irisin (5 nM) (067–29, Phoenix Pharmaceuticals, Inc., Burlingame, CA, USA), and BAIBA (10 µM) (A0324, Tokyo Chemical Industry Co., Ltd., Tokyo, Japan) for 24 hours. After incubation, total RNA was extracted using 200 μL RNA iso plus. Reverse transcription and qPCR protocols are described above.

### Western blotting

HUVECs were cultured using the Endothelial Cell Growth Medium 2 Kit (C-22111, PromoCell, Heidelberg, Germany). The cells were incubated with or without TNFα (10 ng/mL), Irisin (10 nM), and BAIBA (40 µM) for 24 hours. After incubation, proteins extracted from cultured HUVECs were separated by SDS-PAGE and transferred to nitrocellulose membranes. After blocking with 5% bovine serum albumin (BSA) in Tris-buffered saline with 0.1% Tween 20 (TBS-T) for one hour at room temperature, incubated with primary antibodies including anti-VCAM-1 (1:1000, #ab134047, abcam) and anti-MCP-1 (1:1000, #ab9669, abcam) antibodies overnight at 4 °C, washed with TBS-T three times, and incubated with secondary antibody (1:2000, #7074, Cell Signaling Technology Japan K.K., Tokyo, Japan) for one hour at room temperature. The membranes were washed with TBS-T three times and signals were then detected using a chemiluminescence kit (RPN2236, GE Healthcare Japan, Tokyo, Japan), C-DiGit Blot Scanner (LI-COR Biosciences, Lincoln, NE, USA), and Image Studio Software (LO-COR Biosciences).

### Statistical analysis

Statistical analysis was performed using GraphPad Prism (GraphPad Software, Inc., La Jolla, CA, USA). Student’s t-test (for comparisons between two groups) and one-way analysis of variance (ANOVA; for comparisons of three or more groups) followed by Tukey’s range test. All data are expressed as the mean ± SEM.

## Supplementary information

SUPPLEMENTAL MATERIAL

## References

[1] Libby P (2002). Inflammation in atherosclerosis. Nature.

[2] Diep L, Kwagyan J, Kurantsin-Mills J, Weir R, Jayam-Trouth A (2010). Association of physical activity level and stroke outcomes in men and women: a meta-analysis. J. Womens. Health (Larchmt)..

[3] Tanasescu M, Leitzmann MF, Rimm EB, Hu FB (2003). Physical activity in relation to cardiovascular disease and total mortality among men with type 2 diabetes. Circulation.

[4] Shing CM, Fassett RG, Peake JM, Coombes JS (2015). Voluntary exercise decreases atherosclerosis in nephrectomised ApoE knockout mice. PLoS One.

[5] Meilhac O, Ramachandran S, Chiang K, Santanam N, Parthasarathy S (2001). Role of arterial wall antioxidant defense in beneficial effects of exercise on atherosclerosis in mice. Arterioscler. Thromb. Vasc. Biol..

[6] Gielen S, Schuler G, Adams V (2010). Cardiovascular effects of exercise training: Molecular mechanisms. Circulation.

[7] Boo YC (2002). Shear stress stimulates phosphorylation of endothelial nitric-oxide synthase at Ser 1179 by Akt-independent mechanisms. Role of protein kinase A. J. Biol. Chem..

[8] Adams V (2013). Exercise training in patients with chronic heart failure promotes restoration of high-density lipoprotein functional properties. Circ. Res..

[9] Ennezat PV (2001). Physical training in patients with chronic heart failure enhances the expression of genes encoding antioxidative enzymes. J. Am. Coll. Cardiol..

[10] Goto M (2000). cDNA cloning and mRNA analysis of PGC-1 in epitrochlearis muscle in swimming-exercised rats. Biochem. Biophys. Res. Commun..

[11] Miura S (2007). An increase in murine skeletal muscle peroxisome proliferator-activated receptor-γ coactivator-1α (PGC-1α) mRNA in response to exercise is mediated by β-adrenergic receptor activation. Endocrinology.

[12] Puigserver P (1998). A cold-inducible coactivator of nuclear receptors linked to adaptive thermogenesis. Cell.

[13] Tadaishi M (2011). Skeletal muscle-specific expression of PGC-1alpha-b, an exercise-responsive isoform, increases exercise capacity and peak oxygen uptake. PLoS One.

[14] Miura S, Kai Y, Kamei Y, Ezaki O (2008). Isoform-specific increases in murine skeletal muscle peroxisome proliferator-activated receptor-γ coactivator-1α (PGC-1α) mRNA in response to β2-adrenergic receptor activation and exercise. Endocrinology.

[15] Schnyder S, Handschin C (2015). Skeletal muscle as an endocrine organ: PGC-1α, myokines and exercise. Bone.

[16] Steensberg A (2002). IL-6 and TNF-α expression in, and release from, contracting human skeletal muscle. Am. J. Physiol. Endocrinol. Metab..

[17] Carey AL (2006). Interleukin-6 increases insulin-stimulated glucose disposal in humans and glucose uptake and fatty acid oxidation *in vitro* via AMP-activated protein kinase. Diabetes.

[18] Mattson MP, Maudsley S, Martin B (2004). BDNF and 5-HT: A dynamic duo in age-related neuronal plasticity and neurodegenerative disorders. Trends Neurosci..

[19] Matthews VB (2009). Brain-derived neurotrophic factor is produced by skeletal muscle cells in response to contraction and enhances fat oxidation via activation of AMP-activated protein kinase. Diabetologia.

[20] Boström P (2012). A PGC1-α-dependent myokine that drives brown-fat-like development of white fat and thermogenesis. Nature.

[21] Roberts LD (2014). β-aminoisobutyric acid induces browning of white fat and hepatic β-oxidation and is inversely correlated with cardiometabolic risk factors. Cell Metab..

[22] Wang H, Zhang X, Chen W, Huang Q, Chen Q (2015). Relationship between serum irisin levels and urinary albumin excretion in patients with type 2 diabetes. J. Diabetes Complications.

[23] Lee MJ (2015). Irisin, a novel myokine is an independent predictor for sarcopenia and carotid atherosclerosis in dialysis patients. Atherosclerosis.

[24] Zhang SH, Reddick RL, Piedrahita JA, Maeda N (1992). Spontaneous hypercholesterolemia and arterial lesions in mice lacking apolipoprotein E. Science.

[25] Hurtubise J (2016). The different facets of dyslipidemia and hypertension in atherosclerosis. Curr. Atheroscler. Rep..

[26] Nordestgaard BG, Tybjaerg-Hansen A (1992). IDL, VLDL, Chylomicrons and atherosclerosis. Eur. J. Epidemiol..

[27] Ho E, Karimi Galougahi K, Liu C-C, Bhindi R, Figtree GA (2013). Biological markers of oxidative stress: Applications to cardiovascular research and practice. Redox Biol..

[28] Park C (2013). Fasting glucose level and the risk of incident atherosclerotic cardiovascular diseases. Diabetes Care.

[29] Schlitt A (2006). Further evaluation of plasma sphingomyelin levels as a risk factor for coronary artery disease. Nutr. Metab. (Lond)..

[30] Ramji DP, Davies TS (2015). Cytokines in atherosclerosis: Key players in all stages of disease and promising therapeutic targets. Cytokine and Growth Factor Reviews.

[31] Galkina E, Ley K (2007). Vascular adhesion molecules in atherosclerosis. Arterioscler. Thromb. Vasc. Biol..

[32] Deshmane SL, Kremlev S, Amini S, Sawaya BE (2009). Monocyte chemoattractant protein-1 (MCP-1): An overview. J. Interf. Cytokine Res..

[33] Feng Y (2014). TLR4/NF-κB signaling pathway-mediated and oxLDL-induced up-regulation of LOX-1, MCP-1, and VCAM-1 expressions in human umbilical vein endothelial cells. Genet. Mol. Res..

[34] Min J-K (2005). TNF-related activation-induced cytokine enhances leukocyte adhesiveness: induction of ICAM-1 and VCAM-1 via TNF receptor-associated factor and protein kinase C-dependent NF-κB activation in endothelial cells. J. Immunol..

[35] Sukovich DA (1998). Expression of interleukin-6 in atherosclerotic lesions of male ApoE-knockout mice: inhibition by 17beta-estradiol. Arterioscler. Thromb. Vasc. Biol..

[36] Rus HG, Niculescu F, Vlaicu R (1991). Tumor necrosis factor-alpha in human arterial wall with atherosclerosis. Atherosclerosis.

[37] Zhang Y (2016). Protective effect of irisin on atherosclerosis via suppressing oxidized low density lipoprotein induced vascular inflammation and endothelial dysfunction. PLoS One.

[38] Aziz H, Zaas A, Ginsburg GS (2007). Peripheral blood gene expression profiling for cardiovascular disease assessment. Genomic Medicine.

[39] Hansson GK (2005). Inflammation, atherosclerosis, and coronary artery disease. N. Engl. J. Med..

[40] Bennett MR, Sinha S, Owens GK (2016). Vascular smooth muscle cells in atherosclerosis. Circ. Res..

[41] Kleinbongard P, Heusch G, Schulz R (2010). TNFα in atherosclerosis, myocardial ischemia/reperfusion and heart failure. Pharmacol. Ther..

[42] Zhang Y (2014). TNF-α promotes early atherosclerosis by increasing transcytosis of LDL across endothelial cells: Crosstalk between NF-κB and PPAR-γ. J. Mol. Cell. Cardiol..

[43] Pellegrin M (2009). Long-term exercise stabilizes atherosclerotic plaque in apoe knockout mice. Med. Sci. Sports Exerc..

[44] Shimada K (2007). Exercise training reduces severity of atherosclerosis in apolipoprotein E knockout mice via nitric oxide. Circ. J..

[45] Okabe T (2007). Swimming reduces the severity of atherosclerosis in apolipoprotein E deficient mice by antioxidant effects. Cardiovasc. Res..

[46] Eckel RH (2014). 2013 AHA/ACC guideline on lifestyle management to reduce cardiovascular risk: A report of the American College of cardiology/American Heart Association task force on practice guidelines. Circulation.

[47] Libby P, Ridker P, Maseri A (2002). Inflammation and atherosclerosis. Circulation.

[48] Cybulsky MI (2001). A major role for VCAM-1, but not ICAM-1, in early atherosclerosis. J. Clin. Invest..

[49] Öhman MK (2010). Monocyte chemoattractant protein-1 deficiency protects against visceral fat-induced atherosclerosis. Arterioscler. Thromb. Vasc. Biol..

[50] Yang AL, Chen HI (2003). Chronic exercise reduces adhesion molecules/iNOS expression and partially reverses vascular responsiveness in hypercholesterolemic rabbit aortae. Atherosclerosis.

[51] Crissey JM (2015). Divergent role of nitric oxide in insulin-stimulated aortic vasorelaxation between low- and high-intrinsic aerobic capacity rats. Physiol. Rep..

[52] Lu J (2015). Irisin protects against endothelial injury and ameliorates atherosclerosis in apolipoprotein E-Null diabetic mice. Atherosclerosis.

[53] Hatazawa Y (2015). Metabolomic analysis of the skeletal muscle mice overexpressing PGC-1α. PLoS One.

[54] Shimada K, Miyazaki T, Daida H (2004). Adiponectin and atherosclerotic disease. Clinica. Chimica. Acta..

[55] Okamoto Y (2002). Adiponectin reduces atherosclerosis in apolipoprotein E-deficient mice. Circulation.

[56] Mazur-Bialy AI, Bilski J, Pochec E, Brzozowski T (2017). New insight into the direct anti-inflammatory activity of a myokine irisin against proinflammatory activation of adipocytes. Implication for exercise in obesity. J. Physiol. Pharmacol..

[57] Burgomaster KA (2008). Similar metabolic adaptations during exercise after low volume sprint interval and traditional endurance training in humans. J Physiol..

[58] Miyamoto-Mikami E (2015). Endurance training-induced increase in circulating irisin levels is associated with reduction of abdominal visceral fat in middle-aged and older adults. PLoS One.

[59] Piedrahita JA, Zhang SH, Hagaman JR, Oliver PM, Maeda N (1992). Generation of mice carrying a mutant apolipoprotein E gene inactivated by gene targeting in embryonic stem cells. Proc. Natl. Acad. Sci. USA.

[60] Usui S, Hara Y, Hosaki S, Okazaki M (2002). A new on-line dual enzymatic method for simultaneous quantification of cholesterol and triglycerides in lipoproteins by HPLC. J. Lipid Res..

[61] Inoue M (2017). Effects of the dietary carbohydrate–fat ratio on plasma phosphatidylcholine profiles in human and mouse. J. Nutr. Biochem..

[62] Mori K, Kobayashi C, Tomita T, Inatomi S, Ikeda M (2008). Antiatherosclerotic effect of the edible mushrooms Pleurotus eryngii (Eringi), Grifola frondosa (Maitake), and Hypsizygus marmoreus (Bunashimeji) in apolipoprotein E-deficient mice. Nutr. Res..

