# Supplementary material for: Skeletal Muscle-specific PGC-1α Overexpression Suppresses Atherosclerosis in Apolipoprotein E-Knockout Mice
Source: Sci Rep. 2019 Mar 11;9:4077. doi: 10.1038/s41598-019-40643-1 (PMC6411944; doi:10.1038/s41598-019-40643-1)
Supplement: Supplementary file 1 — SUPPLEMENTAL MATERIAL [file 41598_2019_40643_MOESM1_ESM.docx]

SUPPLEMENTAL MATERIAL

**Skeletal Muscle-specific PGC-1α Overexpression Suppresses Atherosclerosis in Apolipoprotein E-Knockout Mice**

Yuki Shimba^a, b^, Hanako Togawa^c^, Nanami Senoo^a,d^, Masahiko Ikeda^e^, Noriyuki Miyoshi^f^, Akihito Morita^a^, Shinji Miura^a*^

^a^ Laboratory of Nutritional Biochemistry, Graduate School of Nutritional and Environmental Sciences, University of Shizuoka, 52-1 Yada, Suruga-ku, Shizuoka 422-8526, Japan.

^b^ Research and Development Department, Tokai Hit Co., Ltd., 306-1 Gendoji-cho, Fujinomiya-shi, Shizuoka 418-0074, Japan.

^c^ Faculty of Social and Environmental Studies, Fuji Tokoha University, 325 Ohbuchi, Fuji-shi, Shizuoka 417-0801, Japan.

^d^ Research Fellow of Japan Society for the Promotion of Science, 5-3-1 Kojimachi, Chiyoda-ku, Tokyo 102-0083, Japan.

^e^ Graduate School of Environment and Disaster Research, Tokoha University, 325 Ohbuchi, Fuji-shi, Shizuoka 417-0801, Japan.

^f^ Laboratory of Biochemistry, Graduate School of Nutritional and Environmental Sciences, University of Shizuoka, 52-1 Yada, Suruga-ku, Shizuoka 422-8526, Japan.

*Corresponding author

Shinji Miura, Ph.D., Laboratory of Nutritional Biochemistry, Graduate School of Nutritional and Environmental Sciences, University of Shizuoka, 52-1 Yada, Suruga-ku, Shizuoka 422-8526, Japan. Email; [miura@u-shizuoka-ken.ac.jp](mailto:miura@u-shizuoka-ken.ac.jp)

**Supplemental Table S1: Primer sequences for qPCR.**

| Genes | Forward primers (5'→3') | Reverse primers (5'→3') |
| --- | --- | --- |
| Mouse *β-actin* | CCTCTATGCCAACACAGTGC | GTACTCCTGCTTGCTGATCC |
| Mouse *eNOS* | TGACCAGCACATTTGGCAATGG | CATGAGCGCTGCTGCAAAGC |
| Mouse *VCAM-1* | TACCAGCTCCCAAAATCCTG | TCTGCTAATTCCAGCCTCGT |
| Mouse *ICAM-1* | GTGATCCCTGGGCCTGGTG | GGAAACGAATACACGGTGATGG |
| Mouse *MCP-1* | CTGGATCGGAACCAAATGAG | AAGGCATCACAGTCCGAGTC |
| Mouse *NFκB* (*p50*) | ATGGCAGACGATGATCCCTAC | CGGATCGAAATCCCCTCTGTT |
| Mouse *NFκB* (*p65*) | ATGTGGAGATCATTGAGCAGC | CCTGGTCCTGTGTAGCCATT |
| Mouse *IL-6* | ACCACGGCCTTCCCTACTTC | CTCATTTCCACGATTTCCCAG |
| Mouse *TNFα* | CAGGCGGTGCCTATGTCTC | CGATCACCCCGAAGTTCAGTAG |
| Mouse *IL-1β* | ACTGTTTCTAATGCCTTCCC | ATGGTTTCTTGTGACCCTGA |
| Mouse *TGF-β* | GTAACGCCAGGAATTGTTGCTA | CTTCAATACGTCAGACATTCGGG |
| Mouse *Acads* | GACTGGCGACGGTTACACA | GGCAAAGTCACGGCATGTC |
| Mouse *Hadha* | TGCATTTGCCGCAGCTTTAC | GTTGGCCCAGATTTCGTTCA |
| Mouse *Hadh* | TCAAGCATGTGACCGTCATCG | TCTCCAGCCTTAGGGTTTTCT |
| Mouse *FNDC5* | ATGAAGGAGATGGGGAGGAA | GCGGCAGAAGAGAGCTATAACA |
| Human *β-actin* | TGGCACCCAGCACAATGA | CTAAGTCATAGTCCGCCTAGAAGCA |
| Human *VCAM-1* | GGGAAGATGGTCGTGATCCTT | TCTGGGGTGGTCTCGATTTTA |
| Human *MCP-1* | CAGCCAGATGCAATCAATGCC | TGGAATCCTGAACCCACTTCT |
| Human *eNOS* | TGATGGCGAAGCGAGTGAAG | ACTCATCCATACACAGGACCC |
| Human *ICAM-1* | ATGCCCAGACATCTGTGTCC | GGGGTCTCTATGCCCAACAA |
| Human *NFkB* (*p50*) | AACAGAGAGGATTTCGTTTCCG | TTTGACCTGAGGGTAAGACTTCT |
| Human *NFκB* (*p65*) | ATGTGGAGATCATTGAGCAGC | CCTGGTCCTGTGTAGCCATT |
| Human *IL-6* | ACTCACCTCTTCAGAACGAATTG | CCATCTTTGGAAGGTTCAGGTTG |

**
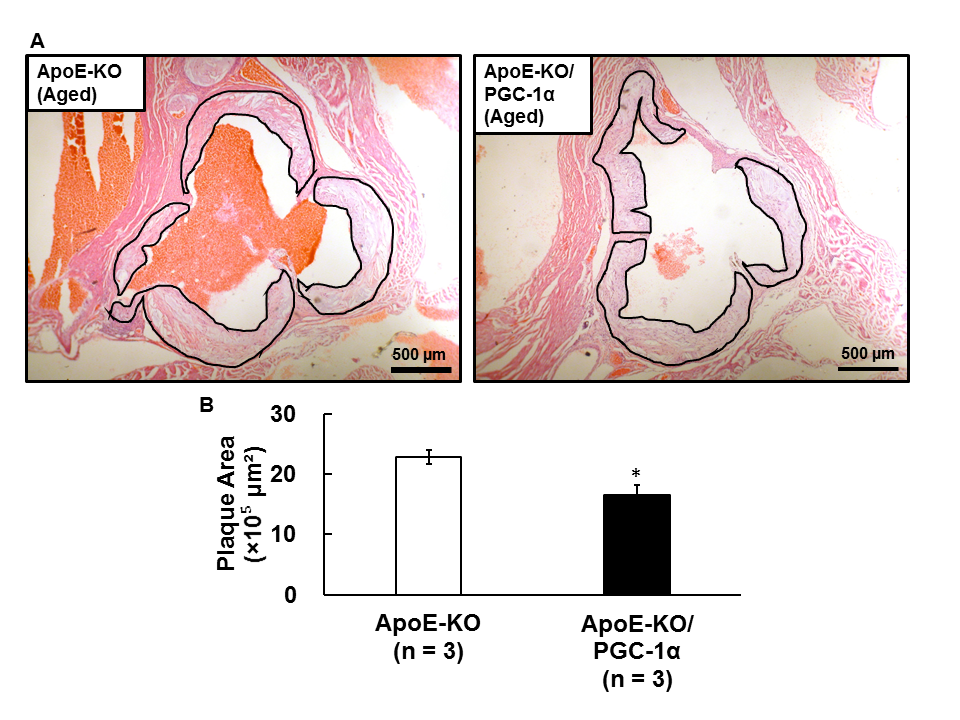
Supplemental Figure S1: Influence of skeletal muscle-specific PGC-1α overexpression on atherosclerotic plaque formation in aged ApoE-KO mice.** (A) Representative images of H&E staining of the aortic valve from aged ApoE-KO and ApoE-KO/PGC-1α mice (37–41 weeks old) are shown. The areas surrounded by a black line indicate atherosclerotic plaques. (B) Quantitation of plaque area. The data are expressed as the mean ± SEM. **p* < 0.05 vs. ApoE-KO mice.

**
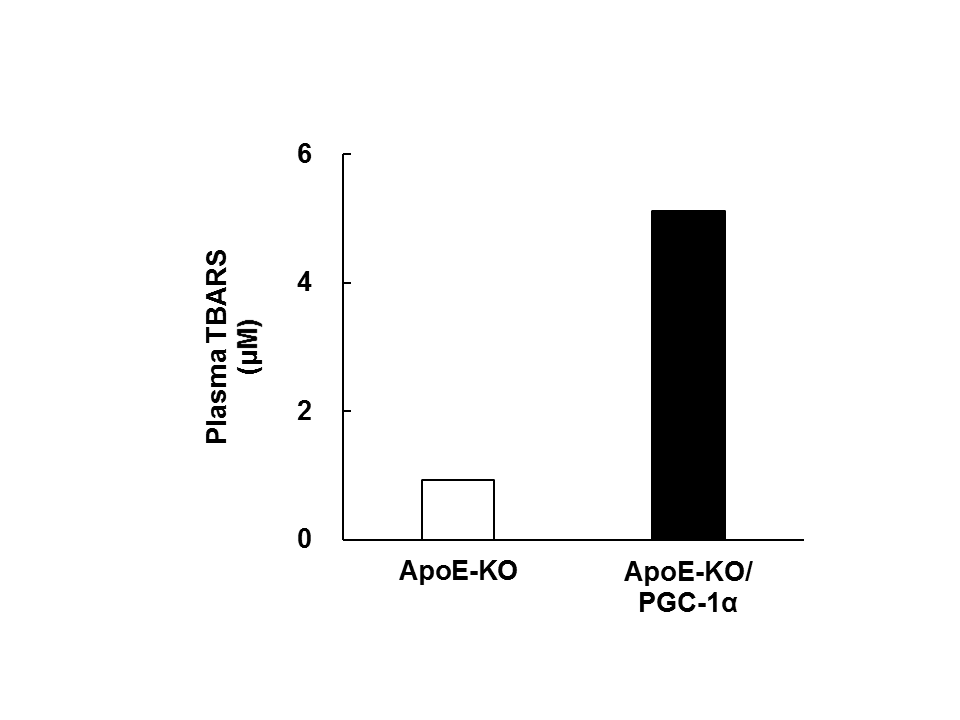
Supplemental Figure S2: TBARS concentration in plasma of ApoE-KO and ApoE-KO/PGC-1α mice.** Plasma of 28 week-old ApoE-KO (n = 4) and ApoE-KO/PGC-1α (n = 3) mice was collected and TBARS concentrations in the pooled plasma were measured. Plasma TBARS concentrations are indicated as malondialdehyde (MDA) equivalents.

**
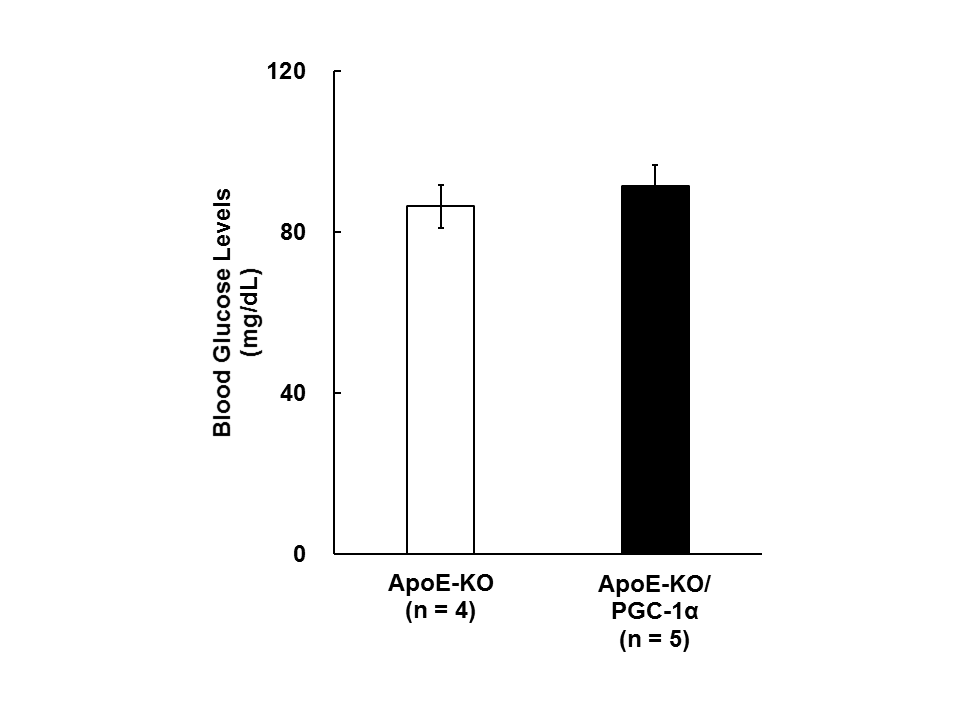
Supplemental Figure S3: Fasting blood glucose levels in ApoE-KO mice and ApoE-KO/PGC-1α mice.** Fasting blood glucose levels of 16–17-week-old mice were measured. The data are expressed as the mean ± SEM.

**
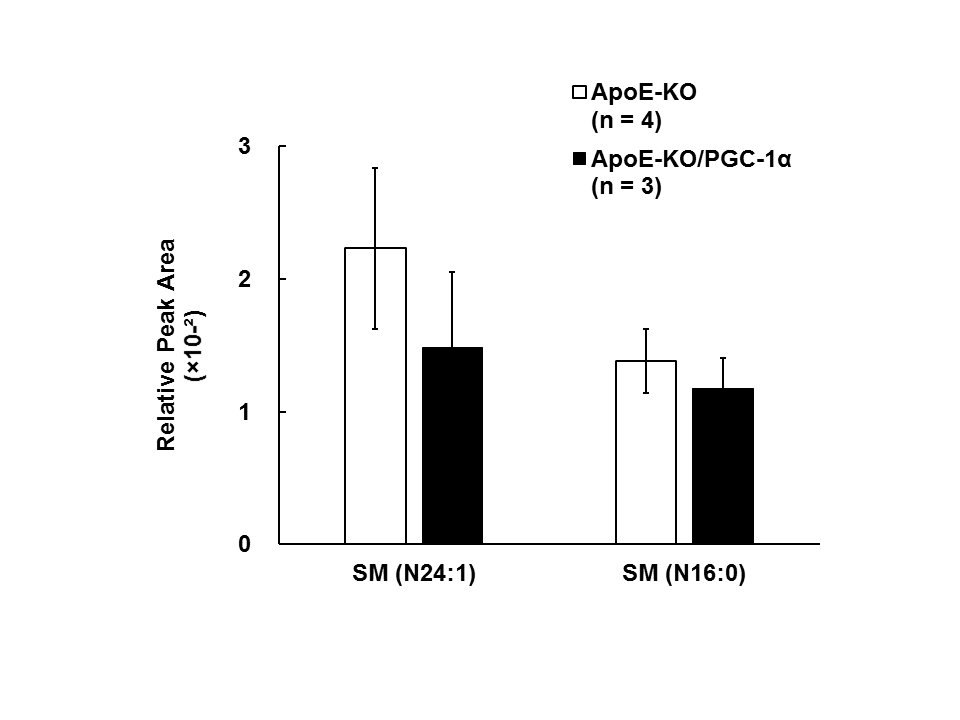
Supplemental Figure S4: Plasma SM levels in ApoE-KO mice and ApoE-KO/PGC-1α mice.** Relative peak areas of major SM (SM (N24:1), SM(N16:0)) in plasma are shown. Two SM species were analyzed using LC/MS. The data are expressed as the mean ± SEM.

**
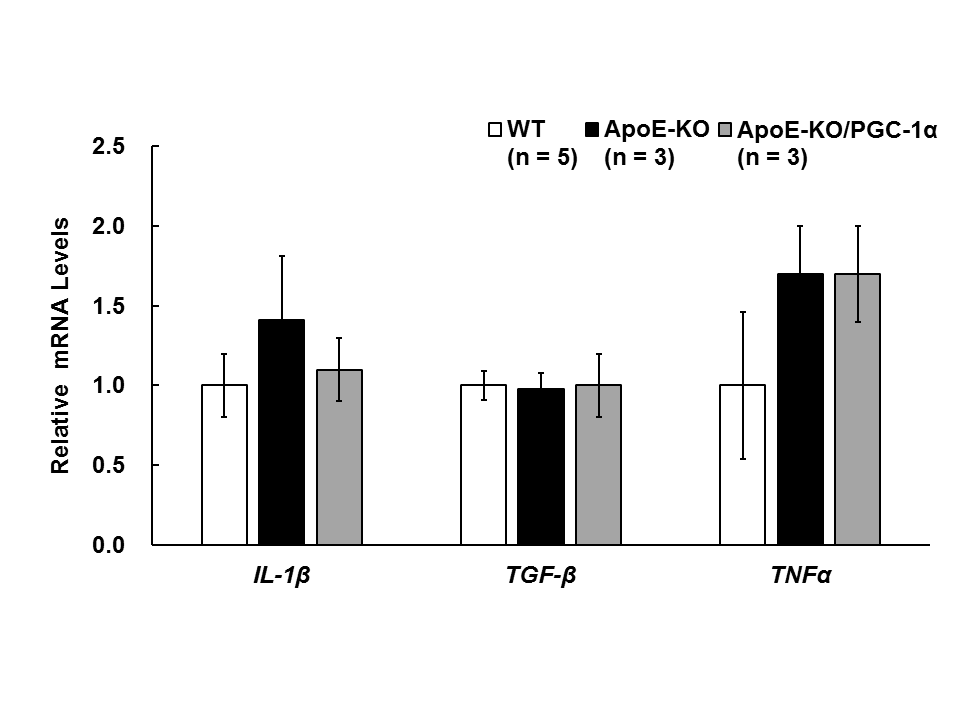
Supplemental Figure S5: mRNA expression of inflammatory mediators in blood cells.** Relative mRNA expression levels of *interleukin-1β* (*IL-1β*), *transforming growth factor-β* (*TGF-β*), and *TNFα* in blood cells were measured by RT-qPCR (3–5 mice in each group). Twenty-eight-week-old mice were used in the experiment. Amplification of *β-actin* mRNA was used to normalize for differences in RNA extraction and amplification. The data are expressed as fold of the mean value in the wild-type (WT) group. Values are mean ± SEM.

**
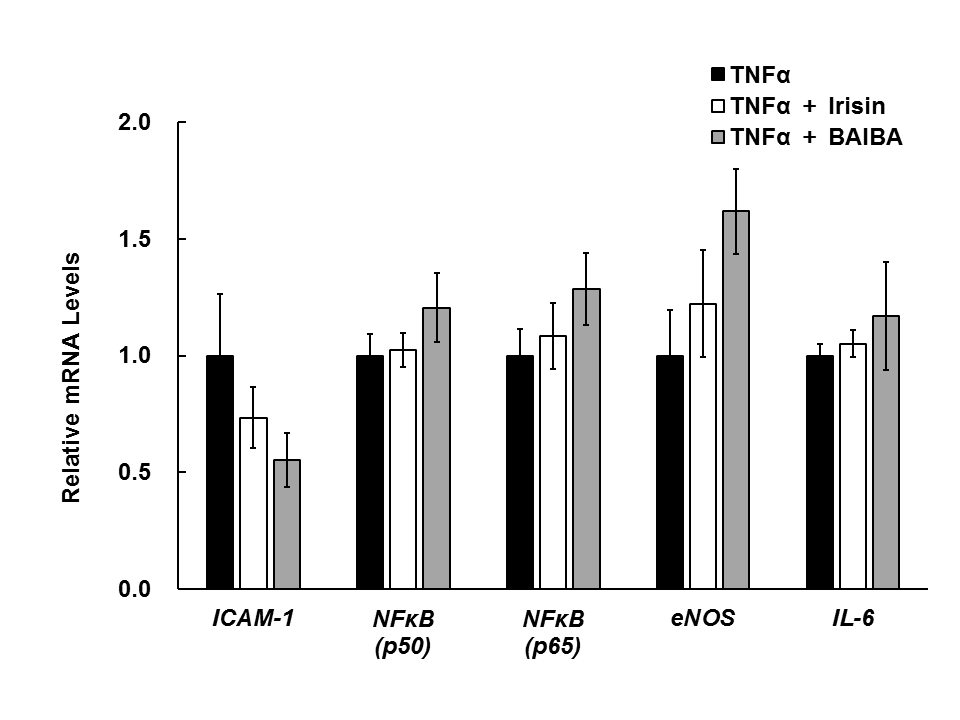
Supplemental Figure S6: Effect of Irisin and BAIBA on TNFα-induced expression of inflammatory mediators in HUVECs.** *eNOS*, *ICAM-1*, *NFκB* (*p50*, *p65*), and *IL-6* gene expression in HUVECs was measured after treatment with Irisin and BAIBA in the presence of TNFα. Expression of these mRNAs was measured by RT-qPCR (n = 6). Amplification of *β-actin* mRNA was used to normalize for differences in RNA extraction and amplification. The data are expressed as fold of the mean value of HUVECs cultured with TNFα. Values are mean ± SEM.

**
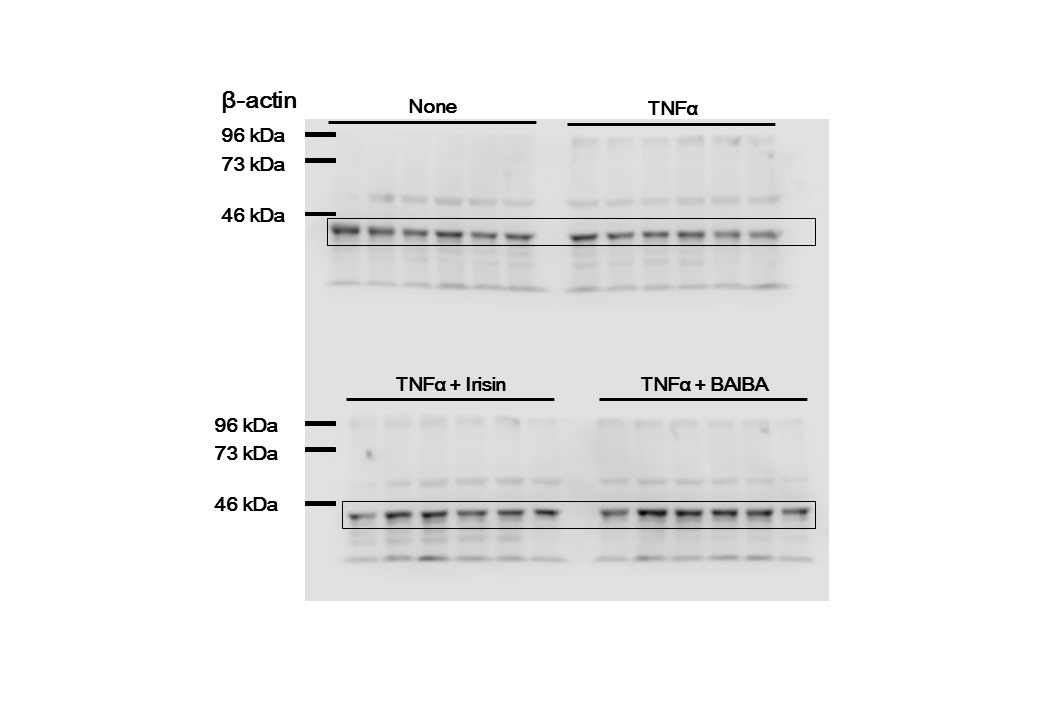
**
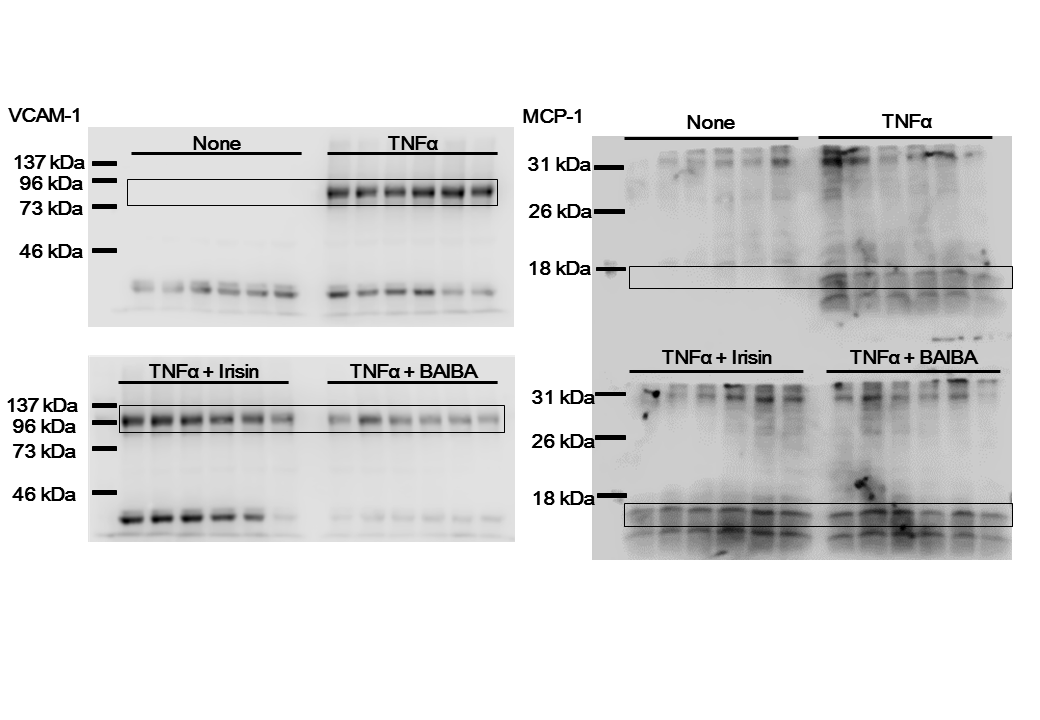
**Supplemental Figure S7: Full-length blot images for cropped western blots (Fig.7).**
